# Effectiveness of Technology-Enabled Knowledge Translation Strategies in Improving the Use of Research in Public Health: Systematic Review

**DOI:** 10.2196/17274

**Published:** 2020-07-31

**Authors:** Alison Brown, Courtney Barnes, Judith Byaruhanga, Matthew McLaughlin, Rebecca K Hodder, Debbie Booth, Nicole Nathan, Rachel Sutherland, Luke Wolfenden

**Affiliations:** 1 School of Medicine and Public Health University of Newcastle Callaghan Australia; 2 Hunter New England Population Health Wallsend Australia; 3 Hunter Medical Research Institute New Lambton Heights Australia; 4 Priority Research Centre for Health Behaviour University of Newcastle Callaghan Australia; 5 University Library Academic Division University of Newcastle Callaghan Australia

**Keywords:** knowledge translation, public health

## Abstract

**Background:**

Knowledge translation (KT) aims to facilitate the use of research evidence in decision making. Changes in technology have provided considerable opportunities for KT strategies to improve access and use of evidence in decision making by public health policy makers and practitioners. Despite this opportunity, there have been no reviews that have assessed the effects of digital technology-enabled KT (TEKT) in the field of public health.

**Objective:**

This study aims to examine the effectiveness of digital TEKT strategies in (1) improving the capacity for evidence-based decision making by public health policy makers and practitioners, (2) changing public health policy or practice, and (3) changes in individual or population health outcomes.

**Methods:**

A search strategy was developed to identify randomized trials assessing the effectiveness of digital TEKT strategies in public health. Any primary research study with a randomized trial design was eligible. Searches for eligible studies were undertaken in multiple electronic bibliographic databases (Medical Literature Analysis and Retrieval System Online [MEDLINE], Excerpta Medica dataBASE [EMBASE], PsycINFO, Cumulative Index to Nursing and Allied Health Literature [CINAHL], and Scopus) and the reference lists of included studies. A hand search of 2 journals (Implementation Science and Journal of Medical Internet Research) and a gray literature search were also conducted. Pairs of independent review authors screened studies, assessed the risk of bias, and extracted data from relevant studies.

**Results:**

Of the 6819 citations screened, 8 eligible randomized trials were included in the review. The studies examined the impact of digital TEKT strategies on health professionals, including nurses, child care health consultants, physiotherapists, primary health care workers, and public health practitioners. Overall, 5 of the interventions were web-training programs. The remaining 3 interventions included simulation games, access to digital resource materials and the use of tailored messaging, and a web-based registry. The findings suggest that digital TEKT interventions may be effective in improving the knowledge of public health professionals, relative to control, and may be as effective as a face-to-face KT approach. The effectiveness of digital TEKT strategies relative to a control or other digital KT interventions on measures of health professional self-efficacy to use evidence to enhance practice behavior or behavioral intention outcomes was mixed. The evidence regarding the effects on changes to health policy or practice following exposure to digital TEKT was mixed. No trials assessed the effects on individual or population-level health outcomes.

**Conclusions:**

This review is the first to synthesize the effectiveness of digital TEKT interventions in a public health setting. Despite its potential, relatively few trials have been undertaken to investigate the impacts of digital TEKT interventions. The findings suggest that although a digital TEKT intervention may improve knowledge, the effects of such interventions on other outcomes are equivocal.

## Introduction

### Background

Investment in public health research is intended to improve public health policy, practice, and decision making [1]. To enhance the impact of public health services, public health care decision making based on high-quality research evidence is recommended [1]. Currently, research evidence is often not utilized optimally in public health decision making [2-4], increasing the risk that public health policies or practices may be enacted that are inferior to alternative interventions, that have little evidence of benefit, or that may be harmful [2,3].

Knowledge translation (KT) is “the synthesis, dissemination, exchange, and application of knowledge in an effort to improve health services and products and strengthen the health care system*”* [5]. The process of KT aims to bridge the gap between research evidence generated by researchers and the use of this evidence by health professionals in their decision making regarding the adoption of health policies, practices, or programs [3,6]. Conceptual frameworks suggest that KT is a dynamic process that is inherently linked to the engagement of end users, including patients, policy makers, and health care professionals, to enhance the uptake of research via the creation, dissemination, and use of knowledge (research) [3]. An important part of the KT processes is the development of knowledge tools, products, or other strategies (*interventions*) that are accessible and interactive and meet the needs of stakeholders for informed decision making [3,4,7].

A variety of factors impede KT in public health. A 2018 critical interpretive synthesis for KT in public health in low- and middle-income countries highlighted the complex nature of creating and accessing evidence for the end user and the contextual factors, including cultural, political, and economic factors that influence the ability to inform evidence-based decision making [8]. A number of KT strategies have previously been applied to address some of these barriers. A systematic review of 5 randomized and non-randomized studies evaluating the effectiveness of public health KT [9] found that single KT strategies, such as disseminating materials to health professionals, were as effective as complex, multicomponent interventions, such as interventions with multiple face-to-face contacts, in changing the practice behavior of public health professionals [9]. However, some KT strategies, such as access to web-based registries to find evidence, did not significantly impact decision making in public health professionals [9].

Changes in technology over recent decades have provided considerable opportunities to improve access and use of evidence in decision making [4]. Technology-enabled KT (TEKT) is the incorporation of digital technology in the application of KT [10]. Digital TEKT does this by using digital technologies, for example, via the use of social media, email, internet, electronic databases, electronic prompts or reminders, web-based webinars, and training or interactive websites. Such strategies may include *push* strategies, whereby research is disseminated (eg, via social media) to target end-user audiences to increase its awareness and use of *pull* strategies that aim to increase the target end user’s demand and use for research (eg, webinars to improve research literacy) [2,11].

Despite the opportunity that digital TEKT presents in facilitating KT, there are few reviews assessing the impact of digital TEKT on decision making in health. To the best of our knowledge, there have been no reviews that have assessed the effects of digital TEKT in the field of public health. This is important given the contextual differences in public health and clinical practice decision making. Nonetheless, reviews of their impact in clinical settings suggest that they can be beneficial. For example, a 2016 systematic review by De Angelis et al [12] examined the impact of information and communication technologies in the dissemination of clinical practice guidelines to health professionals. The review included 21 studies in which dissemination occurred via a variety of communication technologies, including computer software, web-based workshops, educational games, and email and assessed the skills, knowledge, intention of the health professionals, or perceived usefulness of the intervention [12]. The review found that website and computer software dissemination of practice guidelines showed little evidence in improving practice behavior [12]. Conversely, web-based workshops and emails were found to improve practice behavior by improving the skills and knowledge of clinical practice guidelines [12]. Similarly, a 2019 systematic review highlighted that the most effective KT strategies for health professionals in child health settings were those relating to web-based education and computerized prompts and reminders [13]. The existing literature highlights the promise of the use of digital TEKT; however, the effectiveness of digital TEKT in the public health setting is not known.

### Objectives

In the context of existing evidence synthesis gaps for public health digital TEKT, this review aimed to examine the effectiveness of digital TEKT strategies in improving the following measures:

1. Public health policy makers or practitioners’ capacity to make evidence-based health policy and practice decisions such as changes in knowledge acquisition, knowledge retention, change in reasoning, judgment, or decision making [14].
2. Evidence-based public health policy and practice such as changes in behavior, public health policy, or practice.
3. Individual- or population-level health outcomes from the use of research in public health.

## Methods

### Registration

This review was prospectively registered with the International Prospective Register of Systematic Reviews (PROSPERO; CRD42018112715) and is reported per the Preferred Reporting Items for Systematic Reviews and Meta-Analyses guidelines [15].

### Eligibility Criteria

#### Types of Studies

Any primary research study with a randomized trial design, including cluster randomized, was eligible.

Studies published in peer-reviewed journals, as well as gray literature publications, were included in this review. There was no restriction on the length of the study follow-up period, the language of publication, or country of origin.

#### Types of Participants

Studies were eligible for inclusion if they included public health end users, such as health care policy makers, health care managers, and health professionals employed by public health services. For this review, public health services were defined as aiming to promote health and well-being and prevent illness and disease [16]. Such services may be delivered by government or nongovernment organizations. Examples of public health services include health protection and health promotion of preventable diseases and illnesses such as childhood obesity, injury prevention, vaccinations, and immunizations [9,16].

Health professionals who are involved in public health may have included, but was not limited to, health practitioners; allied health professionals such as dietitians, occupational therapists, physiotherapists, speech pathologists; pharmacists; nurses; physicians; and social workers with a focus on preventative care. Studies targeting clinicians were only included if they were engaging in preventative health services. Studies that assessed the impact on researchers embedded within public health services were excluded from this review.

#### Types of Interventions

Any study that reported on the effectiveness of digital TEKT strategies targeting public health policy makers or practitioners was eligible for inclusion that compared the following:

1. Digital TEKT strategies targeting public health policy makers and practitioners with no intervention.
2. Two or more alternative KT strategies targeting public health policy makers and practitioners, where at least one alternative included a digital TEKT strategy only.

Digital TEKT could make use of social media, email, internet, electronic databases, electronic prompts or reminders, web-based webinars, or training or interactive websites to facilitate research use by end users. Digital TEKT strategies could employ either or both *push* and *pull* strategies. Examples of digital TEKT strategies included in this review form part of the knowledge creation component (knowledge tools and products) of the knowledge to action framework [3].

Studies in which digital TEKT was not the exclusive component of a study were excluded.

#### Comparison

Groups may have received any alternative KT strategy, usual care, no intervention, or a waitlist control.

#### Types of Outcome Measures

We included any trial that included the assessment of the effects of digital TEKT strategies on the following:

1. Measures of public health end users’ capacity to make evidence-informed health policy and practice decisions. Any cognitive measures of end users’ capacity for evidence-informed decisions were included, such as measures of knowledge acquisition, knowledge retention, change in reasoning, judgment, or decision making [14]. In addition, measures of change in intention, attitude, and self-efficacy were included. Such data could be collected via surveys of policy makers and practitioners, completion of performance tasks, observations, or other measures.
2. Measures of evidence-based public health policy and practice, including changes in the behavior of policy makers or practitioners in decision making, in actual public health policy, or practice. Measures could include surveys of policy makers and practitioners, practice reviews, and assessment of core competencies or observations.
3. Measures of individual-, community-, or population-level health outcomes. These could include measures of the presence or absence of disease, health condition, or behavioral risk factors (eg, tobacco use) at the level of an individual, collected via patient surveys, use of medical records, objective measures of behavior or disease, or any other method to determine health outcomes; or at a population level, for example, the use of population-level surveys or disease surveillance systems or change in health service use [14].

### Information Sources and Search Methods

A comprehensive search was developed in consultation with an information specialist (DB) conducted for peer-reviewed articles in electronic databases.

#### Electronic Searches

The following electronic databases were searched from inception to October 5, 2018: Medical Literature Analysis and Retrieval System Online (MEDLINE), Excerpta Medica dataBASE (EMBASE), PsycINFO, Cumulative Index of Nursing and Allied Health Literature (CINAHL), and SCOPUS.

We developed the search strategy in MEDLINE and adapted the search for each database (Multimedia Appendix 1). Search filters used in other systematic reviews for KT strategies [9,17,18] and digital dissemination or intervention [12] were adapted for use in this review. Additional search filters were developed to capture the outcomes included in the review [14].

#### Searching for Other Resources

The reference lists of all included studies were searched for additional relevant studies. A hand search of studies published between December 2016 and October 2018 was conducted in the *Implementation Science* and *Journal of Medical Internet Research (JMIR)* journals due to their relevance to the aims of the systematic review. Hand searches were conducted for 2 years, consistent with the practices of other Cochrane Reviews [19,20].

We also conducted a gray literature search using Google, with the search terms “knowledge translation” AND (“digital dissemination OR digital intervention”) AND “public health” and reviewed the first 200 results against the eligibility criteria.

### Selection of Studies

All studies obtained in the literature search were deduplicated. The titles and abstracts of the identified studies were independently screened for eligibility by 2 reviewers (from a pool of 4 reviewers: AB, CB, JB, and MM) and full texts of all relevant or unclear studies were obtained and reviewed against the inclusion criteria using Covidence. Any ambiguity in the inclusion of a study was resolved by discussion or by a third reviewer to reach consensus. Review authors were not blinded to author or journal information.

### Data Collection and Analysis

#### Data Extraction and Management

Two review authors (AB and LW) independently extracted information from the included studies using a data extraction form developed and piloted by the research team. Any discrepancies regarding data extraction between the review authors were resolved via discussion. An attempt to contact study authors occurred if there was insufficient data in the included studies. The following information was extracted: general information (author name, title, date of publication, and country) methods (study design, setting, duration, sample size, and number of experimental conditions), participants (total number and participant characteristics), types of intervention (characteristics of the digital TEKT intervention and comparison components including the type of strategy and modality [website, web-based training, and emails]), type of outcome measures and results (outcomes aligned to the review inclusion criteria, data collection procedure, effect size, and summary data for each intervention group), and information to allow assessment of risk of study bias. Additionally, we extracted information regarding the intervention for each element recommended by the Template for Intervention Description and Replication (TIDieR) checklist. The TIDieR checklist is a 12-item checklist used to report characteristics of interventions in a structured manner. It includes information on why the study was conducted, how the study was conducted, materials used, where it was conducted, frequency of the intervention, and any tailoring or modifications [21]. Such information was not planned in the review protocol and was not used in formal study synthesis but has been included to enhance characterization of the trials.

### Assessment of Risk of Bias

Two reviewers (AB and RH) independently assessed the risk of bias using the *Cochrane Handbook for Systematic Review of Interventions Risk of bias 2.0* tool [22]. Each included study was assessed for the following risks of bias (and rated as *high*, *low*, or *some concerns*): randomization, deviations from the intended intervention, missing outcome data, measurement of the outcome, and free of selective outcome reporting. The overall risk of bias for each study was determined using guidance from the *Cochrane Handbook for Systematic Review of Interventions Risk of bias 2.0* tool [22]. Discrepancies in rating the risk of bias between reviewers were resolved by discussion.

### Data Synthesis

A random effects meta-analysis was planned in the protocol to provide a quantitative assessment of the effects of interventions. However, meta-analysis could not be conducted as outcomes reported were not able to be pooled due to considerable heterogeneity across the included studies. As such, the results are described narratively by synthesizing study findings by outcome. Within the outcome category, synthesis was then undertaken by first synthesizing the findings of studies comparing digital TEKT versus the control group and then by synthesizing the effects of studies comparing digital TEKT strategies versus an alternative intervention.

## Results

### Description of the Included Studies

A total of 6819 citations were screened for eligibility (Multimedia Appendix 2). The full text of 46 studies was obtained to determine eligibility against the review criteria. Of these 46 studies, 8 were included in the review [23-30]. The primary reasons for exclusion of ineligible studies were as follows: non–randomized controlled trials (non-RCTs; n=17), nondigital studies (n=6), not specifically KT (n=6), protocol only (n=4), based on the clinical setting (n=4), or had no digital TEKT outcome (n=1).

A summary of the study characteristics and outcomes of the 8 included studies is provided in Table 1. All 8 studies were RCTs, of which 4 were cluster RCTs [25,27,29,30]. A total of 3 studies were conducted in the United States [23-25], 2 in the Netherlands [26,28], 2 in Canada [27,30], and the remaining study was conducted in China [29].

Health professionals recruited in the included studies comprised nurses [23,26,30], nurse practitioners [23], physicians [23], child care health consultants [24], physiotherapists [30], primary health care workers [29], and public health practitioners [30]. A further study recruited program managers or coordinators of public health departments [27] and the remaining study recruited professionals employed in schools, community agencies, and policy-making bodies [25].

There was variability in the digital TEKT interventions tested. Overall, 5 of the interventions were web-based training programs aimed at improving nutrition and physical activity knowledge in children [24], utilizing smoking cessation guidelines [26], body positivity in children and adolescents [30], physical activity levels in patients with cardiovascular risk factors [28], and improving basic public health services knowledge [29]. The remaining 3 interventions included simulation games to improve communication with patients with mental health disorders [23], access to digital resource materials on substance abuse prevention programs [25], and the use of tailored messaging in comparison with knowledge brokering or a web-based registry concerning healthy body weight promotion in children [27]. Of the 8 included studies, 6 reported using evidence-based information to develop the intervention or as part of the program [23-27,30], including the use of systematic reviews [27], and previous programs and studies [23-26,30]. A full description of the intervention consistent with the TIDieR checklist is provided in Multimedia Appendix 3 [23-30].

The majority of comparison groups for the included studies were control groups that had no access to the digital TEKT strategies (n=3) [23,24,26] or waitlist control group (n=2) [28,30]. A study that aimed at providing information on substance abuse prevention programs compared groups accessing information via CD-ROM or the internet to those who accessed information via printed materials [25]. A further study in public health department personnel compared its most interactive KT strategy (access to knowledge broker, web-based registry, and tailored messages) and its moderate interactive KT strategy (web-based registry and tailored messages) with the least interactive KT strategy of access to a web-based registry only [27]. The study in primary health care workers compared its face-to-face education group with its comparison group that accessed information via websites only, in an effort to improve cardiovascular risk management [29].

The majority of studies reported on fidelity with the intervention to some extent; however, studies were inconsistent in how fidelity was measured, which included time to complete intervention and percent use of program [23,24,27,28,30]. Of the 2 studies that reported on time to complete, the mean time ranged from 124 to 4998 min [23,24] and the percent usage of the program ranged from 45% to 100% in the 3 studies that reported this [27,28,30].

The most commonly reported KT outcome measure was a change in knowledge, as reported in 4 of the included studies [23,24,26,29,30]. Of the 8 included studies, 3 reported the outcome measure of intention to change behavior [23,25,28] and 2 reported self-efficacy for identifying evidence and confidence to improve the health professional’s practice behavior [25,30]. Only 1 study reported the impact of digital TEKT strategies on measures of attitude regarding health professional practice behavior to improve physical activity in cardiovascular patients and on the health professional’s perceived behavior control [28]. Changes to health policy, practice, or decision making were reported by 3 studies [26-28]. No study reported outcomes relating to individual-, community-, or population-level health outcomes.

The majority of outcome measures within the included studies (n=7) were evaluated using telephone, written, or web surveys [23,25-30], with the remaining study using a knowledge test to measure its outcome [24]. Of the 8 included studies, 2 studies reported using validated measures of trial outcomes [23,30].

**Table 1:** Study characteristics and key findings.

Study (reference, country, study design)	Population: type of participant, sample size, mean age and gender of participants, industry experience	Intervention description; duration of intervention; comparator	Outcomes and measures	Key findings
Albright et al [23]**;** United States**;** RCT^a^	227 nurses, nurse practitioners, and physicians; 81.9% female; 65.6% nurses; Industry experience: mean 10.89 (SD 11.01) years; Age not reported	*Description*: At risk in primary care web-based simulation *role-playing* game. Provides learners opportunities to practice role playing with emotionally responsive virtual patients that are experiencing mental health disorders; *Duration*: Simulation takes 1-1.5 hours to complete. *Comparator*: control	*Outcome*: knowledge and skills; *Measure*: 6-item Gatekeeper Behavior Scale web-based questionnaire; *Outcome*: likelihood to screen and manage mental health issues; *Measure*: single item, 4-point Likert-type scale web-based questionnaire	The score for the treatment group, postsimulation (mean 3.40, SD 0.89) was significantly higher than the control group at presimulation (mean 2.91, SD 0.69), *P*<.001; Likelihood of engaging in screening behavior for the treatment group (mean 3.27, SD 0.74) was significantly higher than the control group (mean 2.90, SD 0.87), *P*<.01
Benjamin et al [24]**;** United States**;** RCT	51 CCHCs^b^; Control (n=17): 6.9 years old; 94% female; 88% nursing degree. Web-based (n=17): 41.9 years old; 100% female; 94% nursing degree. In-person (n=16): 39.8 years old; 100% female; 87% nursing degree. Industry experience not reported	*Description*: web-based (group 1) and in-person trained (group 2) CCHCs. Each training included 4 modules: intervention overview, introduction to childhood overweight, nutrition and physical activity, and providing consultation to child care centers. In-person training and web-based training were designed to be similar in both content and structure. *Duration*: training took 3 hours; *Comparator*: control	*Outcome*: nutrition knowledge related to childhood overweight. *Measure*: 28 multiple choice questions (childhood overweight=4, nutrition for children=10, physical activity for children=8, and nutrition and physical activity for adults=6) with 2-5 possible response options	Participants from the web-based trained group (difference in pre/post score=16.18) did not perform better than the in-person trained group (difference in pre/post score=16.53). Both training groups improved significantly more than controls (difference in pre/post score=1.89; *P*<.001 for each group)
de Ruijter et al [26]**;** the Netherlands**;** RCT	269 PNs^c^ across the Netherlands. Mean 47.3 years old; 97.8% female. PN counseling experience was mean 5.6 years	*Description*: Guideline adherence to smoking cessation counseling. Computer-tailored, web-based program relating to smoking cessation. Consisted of web-based modules, tailored advice, forum, and smoking cessation counseling materials. *Duration*: 6 months to access and use the program. *Comparator:* control group engaged in normal smoking cessation counseling practices	*Outcome*: adherence to STIMEDIC^d^ guidelines. *Measure*: questions on guideline adherence concerned the 9 evidence-based counseling steps, as described in the STIMEDIC guideline. PNs adherence at baseline was assessed by asking PNs to self-report their adherence to each guideline step during complete smoking cessation trajectories of their last 10 patients (range 0-10). Additionally, during the trial period, guideline adherence was assessed by asking PNs to self-report their adherence to each guideline step after every consultation with a smoking patient using the counseling checklist	Overall intervention effect not reported. Significant interaction between groups based on the average years of counseling experience (*P*=.045)
Di Noia et al [25]; United States**;** RCT	188 school, community agency, and policy-making professionals; 68.6% females; 25% 30-39 years, 23% 40-49 years, 19% 50-59 years (mean age not reported); 48% some graduate school, 22.3% college, 10.6% some college. Industry experience not reported	*Description*: illustrative dissemination materials for 3 youth-oriented substance abuse prevention programs. Materials for each program were tailored for each setting (school, community agencies, and policy makers) and disseminated by: Group 1: accessed resource materials via CD-ROM (n=64); Group 2: accessed resource materials via the internet (n=69). *Duration*: 2 years. *Comparator*: resource materials accessed via printed pamphlets (n=55)	*Outcome*: self-efficacy. *Measure*^e^: via survey assessing professionals' self-efficacy for identifying and obtaining prevention programs to serve the needs of youth; confidence in ability to recommend programs to their constituents. *Outcome*: intention to apply prevention program materials; likelihood of their future applications of materials disseminated in the trial; likelihood of request program materials, implementing a prevention program and recommending programs to their constituents. *Measure*: via survey	No significant differences between groups for self-efficacy for the ability to identifying programs or recommend programs. Significant between channel effects in ability to obtain programs for pamphlet (mean 1.37, SD 0.93) versus internet (mean 0.87, SD 0.79) and pamphlet versus CD-ROM (mean 0.94, SD 0.84) at *P*<.05 at follow-up; No significant differences between groups for likelihood of requesting program or likelihood of implementing program. Significant difference between CD-ROM (mean 1.41, SD 1.13) and pamphlet (mean 1.55, SD 1.13) for likelihood of recommending program at *P*<.05 and significant difference between CD-ROM and internet (mean 1.06, SD 1.05) for likelihood of recommending program at *P*<.05 at follow-up
Dobbins et al [27]**;** Canada**;** RCT	108 public health departments with program managers and/or coordinators and/or program directors responsible for making program decisions related to healthy body weight promotion in children; 35% frontline staff; 26% manager; 47% nursing discipline; Mean 5 years in current position; Mean years in a public health role=13. Age and gender not reported	*Description*: the 3 interventions included access to a web-based registry of research evidence, tailored messaging, and a knowledge broker. Moderate interactive intervention (digital TEKT^f^ strategy): tailored targeted messages plus access to a health evidence repository (TM). Most interactive intervention: access to a knowledge broker, tailored targeted messages plus access to health evidence repository (KB). Least interactive intervention: access to health evidence repository (HE). *Duration*: program implemented over 1 year	*Outcome*: public health policies and programs; *Measure*: This measure was derived as the sum of actual strategies, policies, and/or interventions for health body weight promotion in children being implemented by the health department. Participants were asked whether the public health policies and programs were being implemented by their health department (yes/no); *Outcome*: global evidence-based decision making. *Measure*: in a telephone-administered survey, participants were asked to report on the extent to which research evidence was considered in a recent program planning decision (previous 12 months) related to healthy body weight promotion	TM group improved significantly from baseline to follow-up in comparison to the HE and KB groups that showed no significant change (*P*<.01); Intervention had no significant effect on global evidence-based decision making (*P*<.45), although all groups improved to some extent (HE group: 0.74; TM group: −0.42; KB group: −0.09)
McVey et al [30]**;** Canada**;** RCT	89 public health practitioners (100% female) from 2 Canadian provinces; Public health participants: public health nurses (n=62) and nutritionists (n=27), with average number of years 12.72; 84.4% identified as white. Age not reported	*Description*: the student body Promoting Health at Any Size web-based program; 6 learning modules: (1) media and peer pressure (2) healthy eating, (3) active living, (4) teasing, (5) adult role models, and (6) school climate including case studies, background information, additional resources, and classroom activities. *Duration*: the intervention group had 60-day access to the web-based intervention. *Comparator*: waitlist control	*Outcome*: knowledge of the physical changes associated with puberty, facts concerning restrictive dieting, peer and adult influences, and the influence of the media on weight loss. *Measure*: assessed via a survey using true-false questions and Likert scales; *Outcome*: efficacy to fight weight bias. *Measure*: 6-item subscale used to assess self-efficacy expectations for fighting weight bias in their schools. On the basis of a 4-point Likert scale (Cronbach α=.44)	Physical changes associated with puberty: there was no significant interaction effect, *F*_1,77_=.486, *P*=.488. Facts concerning restrictive dieting: there were no significant interactions or time effects found for any of the items that tapped knowledge about dieting. Peer or adult influences: there were no significant interactions or time effects. Influence of the media on weight loss: there were no significant interactions or time effects; There was a significant interaction effect found for the variable efficacy to fight weight bias, *F*_1,77_=10.81, *P*=.002. Participants in the intervention group only reported significant improvements in efficacy scores between baseline and the postintervention periods, *P*<.001
Sassen et al [28]; the Netherlands**;** RCT	69 health care professionals with at least a bachelor’s degree in nursing or physiotherapy and who had regular consultations with patients with CV^g^ risk factors and low levels of PA. Control group: 78% female, mean 39.7 years old; 68% bachelor's degree, mean 9.58 years professional experience. Intervention group: 69% female, mean 38.6 years old; 79% bachelor's degree, mean 9.76 years professional experience	*Description*: web-based intervention to increase health care professionals’ intention and encouraging behavior toward patient self-management, following CV risk management guidelines. Website contained modules to help the health professionals improve their professional behavior, support the health professional, improve patients' intention, and risk reduction. The website also included a forum directed at health professionals to share experiences with other professionals. *Duration*: not reported. *Comparator*: waitlist control	*Outcome*: intention to encourage CV patients to become physically active. *Measure*: self-assessed through a questionnaire (3 items on intention); *Outcome*: attitude to encourage PA^h^ in CV patients. *Measure*: self-assessed through a questionnaire of a series of 8 questions regarding the usefulness of assessing patients’ motivation, pros and cons of PA, teaching patients : resisting social pressure, teaching specific PA skills, teaching patients how to handle barriers, formulating PA goals, teaching patients to handle relapses, and helping patients understand the relationship between health problems and PA; *Outcome*: perceived behavior control. *Measure*: self-assessed through a questionnaire (23 items on behavior outcomes); *Outcome*: behavior change in encouraging CV patients to PA. *Measure*: assess via 2 items by asking whether professionals encourage CV patients to increase PA and how often do they encourage CV patients to become physically active	No significant differences in both the intervention and the control groups between baseline (mean: 6.25, SD: 1.00 and mean: 5.87, SD: 1.15) and follow-up (mean: 6.06, SD: 1.11 and mean: 6.02, SD: 091) for intention
				No significant differences in both the intervention and the control groups between baseline (mean: 6.30SD: 0.44 and men: 6.23, SD: 0.69) and follow-up for attitude (mean: 6.30 SD:0.56 and mean: 6.31SD: 0.68); Significant difference in perceived behavior control between baseline and follow-up for the intervention group (t_26_=−2.954, *P*<.001, effect size=0.50) and a significant increase for the control group (t_19_=−2.651, *P*=.02, effect size=0.54). No significant difference between intervention and control group; No significant differences in both the intervention and the control groups between baseline (mean 4.54, SD: 1.02 and mean: 4.83 SD: 0.69) and follow-up for behavior (mean: 4.63, SD: 0.85 and mean:4.79, SD: 0.82)
Zhan et al [29]**;** China**;** RCT	1237 primary health care workers. Blended learning group (n=569): Mean 41.67 years old, 48.9% female, 9.6% technical secondary school or below; Pure web-based learning group (n=563): mean 41.98 years old, 43.2% female, 77.3% technical secondary school or below	*Description*: the blended learning (intervention) and pure web-based learning (control) groups had the same course materials to improve basic public health services knowledge. Participants in the blended learning group studied PowerPoint-based theoretical materials, received the handouts of case study materials for self-studying and attended 1-day (8 hour) face-to-face case study training. *Duration*: overall study period was 5 weeks. *Comparator*: control (pure web-based learning group–digital TEKT strategy); received via a web-based platform: Microsoft PowerPoint; case studies consisted of 3 video sessions, and 2 discussion forums were developed on the training platform	*Outcome*: knowledge for course module components. *Measure*: a total of 3 knowledge MCQ^i^ tests were developed, consisting of a 10-item MCQ test in course module 1, a 15-item MCQ test in course module 2, and a 20-item MCQ test in course module 3	Baseline knowledge scores of the 3 course modules between experimental and control group were similar. Higher gains in the experimental group than in the control group; module 1: adjusted mean difference=4.92, *P*<.001; module 2: adjusted mean difference=3.67, *P*=.004; module 3: adjusted mean difference=4.63, *P*<.001

^a^RCT: randomized controlled trial.

^b^CCHC: child care health consultants.

^c^PN: practice nurses.

^d^STIMEDIC: A registered trademark that stands for smoking cessation (SMR) in health care.

^e^Lower scores indicate more favorable ratings.

^f^TEKT: technology-enabled knowledge translation.

^g^CV: cardiovascular.

^h^PA: physical activity.

^i^MCQ: multiple choice questions.

### Risk of Bias

**Figure 1:**
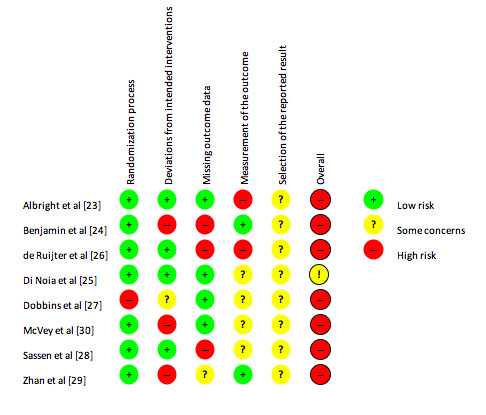
Risk of bias summary.

**Figure 2:**
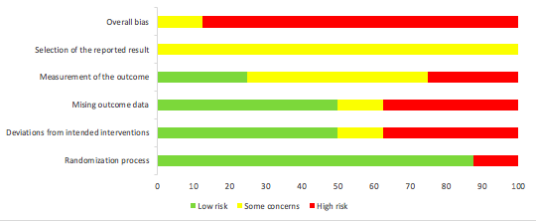
Risk of bias graph.

Figures 1 and 2 summarize the risk of bias for each of the studies.

Of the 8 studies, 7 were assessed as having an overall high risk of bias [23,24,26-30]. Only 1 study was considered to have a high risk of bias for the randomization process [27]. For deviations from intended interventions, 3 studies were assessed as having a high risk of bias [24,29,30], with 1 study having some concerns [27]. Missing outcome data resulted in 3 studies having a high risk of bias [24,26,28], with measurement of the outcome highlighting 2 studies with a high risk of bias [23,26]. All 8 studies were classified as having some concerns in relation to the selection of the reported results.

### Key Findings

A summary of the outcomes and key findings is provided in Table 1.

#### Measures of Public Health End Users’ Capacity to Make Evidence-Informed Decisions

##### Knowledge Change

A total of 4 trials examined the effects of digital TEKT strategies on knowledge outcomes [23,24,29,30]. Of these, 3 compared the effects of digital TEKT interventions with a control, each of which reported significant improvements in knowledge favoring the intervention [23,24,29]. A study using a web-based simulation game reported significant improvements in knowledge and skill scores postintervention to enhance the assessment of mental health disorders (*P*<.001) [23]. Similarly, a study in child care health consultants reported significant improvement in nutrition and physical activity knowledge among those receiving web-based training relative to those in a control that did not provide training (*P*<.001) [24]. A study in primary health care workers in rural China compared the provision of web-based learning only to improve knowledge regarding basic public health services in rural China with a blended learning group provided with a combination of digital and face-to-face training. The study found greater improvements in knowledge of public health services in the blended learning group (*P*<.001) [29]. Finally, a study allocating public health professionals to receive a web-based program to prevent eating disorder behavior in children or a waitlist control reported no significant differences across any knowledge outcomes [30].

Only 1 trial compared the effects of a digital TEKT intervention with an alternate KT intervention [24]. The study of child care health consultants compared nutrition knowledge among those receiving face-to-face training versus those receiving web-based training [24]. The study found no differences between groups in terms of knowledge outcomes.

##### Self-Efficacy

Overall, 2 trials assessed self-efficacy for identifying evidence and confidence to improve the health professional’s practice behavior as a digital TEKT outcome, the findings of which were mixed [25,30]. The first, a study by Mcvey et al [30], found that public health professionals who had accessed the web-based program had significant improvements in self-efficacy scores compared with controls (*P*=.002). The second study compared alternate KT interventions and found no difference in professionals from schools, community agencies, and policy-making bodies, confidence to recommend suitable programs, or self-efficacy for identifying and recommending relevant programs between those allocated to receive substance abuse prevention program resources via CD-ROM or websites compared with those receiving such information via pamphlets [25]. However, there were between-group differences in self-efficacy to obtain youth prevention programs, with those in the internet group and the CD-ROM group reporting significant improvement when compared with the pamphlet group (*P*<.05) [25]. Bonferroni post hoc analyses highlighted favorable differences for the internet group (*P*<.05).

##### Intention

In all 3 studies that measured behavioral intentions reported mixed effects of digital TEKT interventions on these outcomes [23,25,28] and 2 studies compared digital TEKT interventions versus no intervention control. The first, a study by Albright et al [23], found that participants allocated to the web-based simulation game reported a higher likelihood of assessing and screening patients for mental health disorders relative to the control group (*P*<.01). However, Sassen et al [28] reported no significant differences between the website-trained group and the control group in the intention of health professionals to encourage cardiovascular patients to become physically active [28].

The study by Di Noia et al [25] compared the effects of different KT interventions for a substance abuse prevention program. The study found that participants receiving dissemination materials via the internet had significant improvements in their likelihood of recommending programs compared with the CD-ROM group (*P*<.05). The CD-ROM group, however, showed significant improvements compared with the pamphlet group (*P*<.05) [25]. There were no differences between any of the 3 groups in their likelihood of requesting programs or the likelihood of implementing the program [25].

##### Attitude

Only 1 study reported attitude as an outcome measure and reported that there were no differences between the website-trained group and the control group in their attitude to encourage cardiovascular patients to become physically active [28].

##### Perceived Behavior Control

Perceived behavior control to assess health professionals’ perceived skills and knowledge in encouraging physical activity in cardiovascular patients was reported by Sassen et al [28], who found no significant difference between the website-trained group and the control group [28].

#### Measures of Evidence-Based Public Health Policy and Practice

##### Changes to Health Policy, Practice or Decision Making

Of the 8 included studies, 3 reported changes to health policy, practice, or decision making as an outcome measure [26-28]. A web-based study to improve the behavior of health professionals who were nurses or physiotherapists in following cardiovascular risk management guidelines reported no significant differences between groups in encouraging cardiovascular patients to increase physical activity levels (*P*=.68) [28].

A study by de Ruijter et al [26] in practice nurses reported on the outcome of smoking cessation guideline adherence and found that there was a significant difference between groups as counseling experience increased guideline adherence; however, the overall intervention effect was not reported.

The study by Dobbins et al [27] in public health departments reported outcome measures relating to a change in public health policies and programs and a change in global evidence-informed decision making. In relation to public health policies and programs, the group that received tailored messages via email plus access to a health repository improved significantly in the number of public health policies and programs implemented in comparison with both the group that received only access to the health repository and the group that received access to the health repository, tailored messages, and a knowledge broker (*P*<.01) [27]. The study reported no significant differences between groups in relation to global evidence-informed decision making [27].

#### Measures of Individual-, Community-, or Population-Level Health Outcomes

No included studies reported on individual-, community-, or population-level health outcomes.

## Discussion

### Principal Findings

To the best of our knowledge, this review is the first to report on the effectiveness of digital TEKT strategies in improving public health end users’ capacity to make evidence-informed health policy and practice decisions. Overall, the findings from the 8 included studies suggest that digital TEKT interventions may be effective at improving public health professionals’ knowledge, and may be as effective at improving knowledge as a face-to-face KT approach. The effectiveness of digital TEKT strategies relative to control or other KT interventions for self-efficacy or behavioral intention outcomes and changes to health policy or practice were mixed. Such findings offer little guidance for those interested in utilizing digital TEKT strategies to promote the transfer of knowledge to improve public health and demonstrate a considerable need for further research in this field.

The small number of trials identified in this review examined the impact of the digital TEKT intervention on a narrow range of potential outcomes, namely knowledge, self-efficacy, and behavioral intentions. Other outcomes suggested by evaluation frameworks are important in assessing digital TEKT interventions, including behavioral and population-level health outcomes; however, these were rarely reported [14]. These findings are consistent with other systematic reviews of KT and digital dissemination strategies. For example, a systematic review published in 2012 on the effects of KT on public health identified only 5 included studies and reported outcomes relating to knowledge change and change in practice only [9]. Similarly, a systematic review in 2016 including 21 studies on the effectiveness of digital dissemination of clinical practice guidelines included outcomes such as usability of the technology (perceived usefulness and perceived ease of use) and practice behavior (using the theoretical domains framework including knowledge, skills, beliefs, and motivations), with knowledge and skills being the most frequently reported outcome and limited studies reporting other behavior change outcomes [12].

A 2019 systematic review on KT strategies in the clinical child health setting included 48 studies and reported outcomes relating to health care professionals or patient-reported outcomes [13]. Although the review included some digital TEKT studies, only 9 RCTs had a positive effect on clinician-related or patient outcomes [13]. This recent systematic review highlights the continued paucity of research on digital TEKT broadly. The limited reporting of behavioral and population-level health outcomes and the differences in measures used to report other outcomes highlight the challenges of conducting and synthesizing the effects of KT research. Although a variety of tools have been suggested to be useful in assessing the impact of digital KT strategies, including altmetric scores, measures of engagement and process indicators, and use of consistent evaluation metrics in reporting KT outcomes, would assist in appropriately assessing the effectiveness of digital TEKT and comparing these across trials. Core outcome sets are currently being used in several specific health areas to determine a standardized set of outcomes that should be measured and reported [31]. However, there are currently no core outcome sets developed for KT [32]. To address the issue of inconsistent evaluation outcomes in KT research, it may be necessary to develop KT-specific core outcome sets.

For knowledge outcomes, the findings of this review are broadly comparable with other systematic reviews of the effects of KT interventions in other health professions. For example, a 2012 review of public health KT strategies suggested that KT strategies were effective in improving knowledge outcomes, with 2 of 3 included studies reporting significant improvements in measures of knowledge acquisition [9]. Similarly, a 2016 systematic review found improvements in knowledge following web-based workshops, emails, educational web-based games, and multifaceted KT interventions, but not interventions using websites or computer software [12]. Consistent with the findings of this review, other systematic reviews have also reported mixed effects of KT on measures of behavioral intentions [12]. The findings of the review on other outcomes are difficult to contextualize, given the limited number of trials reporting these outcomes. Collectively these findings suggest that digital TEKT strategies may influence precursors for behavior change, such as knowledge and intention, but are yet to demonstrate impacts on public health policy and community outcomes. This is an important evidence gap for researchers to remedy, as public health policy and community impacts represent the ultimate goal of TEKT interventions. Indeed, such outcomes are required for TEKT strategies to yield improvements in public health decision making. The conduct of rigorous trials of digital TEKT strategies that include policy or community health outcomes, however, may represent a considerable challenge for researchers given the limited resources available to conduct such large trials with long periods of follow-up required for KT to occur [3]. Embedding such trials into public health services, which have an interest in KT for health service improvement, and have existing infrastructure and access to routinely collected data on community health improvement may represent a means of addressing the logistical challenges of undertaking such trials [33].

### Strengths and Limitations

There are a number of strengths to this review. A comprehensive search strategy was used in consultation with an information specialist, using multiple electronic databases, hand search of reference lists, and gray literature searches. A systematic approach was utilized to review the current literature on digital TEKT in public health, including the use of pairs of reviewers to double-screen studies for inclusion, extract data, and determine the quality of the studies included. However, there were some limitations that must be considered when interpreting the findings of the review. All but 1 study was assessed as having an overall high risk of bias, particularly due to the measurement of the outcome and selection of the reported results. As most studies had follow-up periods <12 months, it is difficult to determine the effects of the intervention over a longer period of time, which is particularly important when considering behavior and policy change. There was also a high level of heterogeneity due to differing health professional populations, interventions, and outcome measures, making it difficult to draw comparisons between studies and limiting the ability to conduct a meta-analysis. The KT search terms used in this review may have missed relevant KT studies, given the high variability of terms that are used in health to describe KT, a common limitation described in the literature [2,3].

### Conclusions

This review addresses an important knowledge gap for digital TEKT in the public health setting and is the first to synthesize the effectiveness of digital TEKT interventions. Although the review has highlighted potential improvements in knowledge using digital TEKT strategies, it remains unclear whether digital TEKT strategies improve other behavioral and population health outcomes. Currently, there are limited studies assessing digital TEKT in the public health setting, with a limited range of outcomes to assess their effectiveness appropriately. As such, the findings of the review provide limited guidance to assist in the development of effective digital TEKT strategies. A recent scoping review conducted in 2020 summarized the most relevant KT frameworks for use by researchers, policy makers, and clinicians in the health care setting [34]. In the absence of sufficient evidence on the effectiveness of TEKT, the use of relevant KT frameworks is suggested to appropriately disseminate research findings to influence evidence-informed health policy and practice decisions.

## Appendix

Multimedia Appendix 1 Medline Search terms.

Multimedia Appendix 2 PRISMA Flow Diagram.

Multimedia Appendix 3 Template for Intervention Description and Replication checklist for all included studies.

## Abbreviations

CINAHL: Cumulative Index of Nursing and Allied Health Literature
EMBASE: Excerpta Medica dataBASE
JMIR: Journal of Medical Internet Research
KT: knowledge translation
MEDLINE: Medical Literature Analysis and Retrieval System Online
NHMRC: National Health and Medical Research Council
RCT: randomized controlled trial
TEKT: technology-enabled knowledge translation
TIDieR: Template for Intervention Description and Replication
TRIP: Translating Research Into Practice
