# Supplementary material for: Effectiveness of Technology-Enabled Knowledge Translation Strategies in Improving the Use of Research in Public Health: Systematic Review
Source: J Med Internet Res. 2020 Jul 31;22(7):e17274. doi: 10.2196/17274 (PMC7428911; doi:10.2196/17274)
Supplement: Multimedia Appendix 1 [file jmir_v22i7e17274_app1.docx]

### Multimedia Appendix I: Medline Search terms

Database(s): **Ovid MEDLINE(R) and Epub Ahead of Print, In-Process & Other Non-Indexed Citations and Daily**1946 to October 05, 2018 
Search Strategy:

| **#** | **Searches** | **Results** |
| --- | --- | --- |
| 1 | Knowledge translation.tw. or Translational Medical Research/ | 10281 |
| 2 | (Knowledge adj (broker* or transfer or uptake or utili?ation or acquisition or retention)).tw. | 3397 |
| 3 | (Research adj3 (translat* or implement* or transfer* or uptake* or utili?ation)).tw. | 20480 |
| 4 | Disseminat*.tw. | 111717 |
| 5 | Information Dissemination/ | 14650 |
| 6 | Implementation.tw. | 200291 |
| 7 | Professional practice.tw. | 4370 |
| 8 | Professional adherence.tw. | 10 |
| 9 | Practice research.tw. | 3585 |
| 10 | Decision making/ or decision making.tw. | 163424 |
| 11 | 1 or 2 or 3 or 4 or 5 or 6 or 7 or 8 or 9 or 10 | 503889 |
| 12 | digital.tw. | 105518 |
| 13 | online.tw. | 85910 |
| 14 | electronic.tw. | 198031 |
| 15 | (telehealth or tele health).tw. | 3084 |
| 16 | Internet/ | 66011 |
| 17 | Mobile applications/ | 3405 |
| 18 | ("e health" or ehealth or electronic* health).tw. | 14781 |
| 19 | ("m health" or mhealth or mobile health).tw. | 3182 |
| 20 | Medical informatics/ | 10799 |
| 21 | Health information exchange/ | 640 |
| 22 | Website*.mp. | 21623 |
| 23 | Social media/ or (facebook* or youtube or twitter* or webmd or linkedin or medeley or refworks or endnote or Skype or researchgate).tw. | 8842 |
| 24 | Electronic mail/ or (email or "e-mail").tw. | 11979 |
| 25 | ("E learning" or elearning).tw. | 2152 |
| 26 | Digital libraries.tw. | 102 |
| 27 | Reminder systems/ or computeri?ed reminder*.tw. | 3183 |
| 28 | ((online or digital) adj (seminar* or webinar* or workshop* or conference*)).tw. | 51 |
| 29 | Audiovisual aids/ or audiovisual.tw. | 9806 |
| 30 | Telecommunications/ | 4692 |
| 31 | 12 or 13 or 14 or 15 or 16 or 17 or 18 or 19 or 20 or 21 or 22 or 23 or 24 or 25 or 26 or 27 or 28 or 29 or 30 | 484238 |
| 32 | Public health/ or public health.tw. | 238113 |
| 33 | Community health/ or community health.tw. | 93362 |
| 34 | Health promotion/ or Health Services Accessibility/ | 132006 |
| 35 | Health service*.tw. | 96245 |
| 36 | 32 or 33 or 34 or 35 | 445702 |
| 37 | Random allocation/ or random*.tw. | 1053489 |
| 38 | Experiment*.mp. | 2124019 |
| 39 | Intervention*.mp. | 882286 |
| 40 | Randomised controlled trials as topic/ or Clinical trial as topic/ or Randomized controlled trial/ | 635132 |
| 41 | Systematic review.tw. | 115613 |
| 42 | Meta analysis as topic/ | 16444 |
| 43 | (Meta analy* or metanaly* or metaanaly or meta regression).tw. | 134923 |
| 44 | Controlled clinical trial/ | 92676 |
| 45 | Trial.tw. | 517056 |
| 46 | Groups.tw. | 1863589 |
| 47 | 37 or 38 or 39 or 40 or 41 or 42 or 43 or 44 or 45 or 46 | 5534981 |
| **48** | **11 and 31 and 36 and 47** | **2053** |
