# Supplementary material for: Effectiveness of Technology-Enabled Knowledge Translation Strategies in Improving the Use of Research in Public Health: Systematic Review
Source: J Med Internet Res. 2020 Jul 31;22(7):e17274. doi: 10.2196/17274 (PMC7428911; doi:10.2196/17274)
Supplement: Multimedia Appendix 2 [file jmir_v22i7e17274_app2.docx]

### Multimedia Appendix II: PRISMA Flow Diagram

Additional records identified through other sources
(n = 739)

Key reference lists (n=539)

Grey literature search (n=200)

Studies included in qualitative synthesis
(n =8)

Full-text articles assessed for eligibility
(n = 46)

Records excluded
(n = 6773)

Records screened
(n = 6819)

Records after duplicates removed
(n =6819)

## Identification

## Eligibility

## Included

## Screening

Records identified through database searching
(n = 6878)

Full-text articles excluded, with reasons
(n =38)

Non-RCTs (n=17)

Non-TEKT study (n=6)

Not knowledge translation (n=6)

Clinical setting (n=4)

Protocol only (n=4)

No TEKT Outcome (n=1)
