# Supplementary material for: Effectiveness of Technology-Enabled Knowledge Translation Strategies in Improving the Use of Research in Public Health: Systematic Review
Source: J Med Internet Res. 2020 Jul 31;22(7):e17274. doi: 10.2196/17274 (PMC7428911; doi:10.2196/17274)
Supplement: Multimedia Appendix 3 [file jmir_v22i7e17274_app3.docx]

### Multimedia Appendix III: TIDieR Checklist for all included studies

|  | Albright et al [23]**;** United States | Benjamin et al [24]**;** United States | de Ruijter et al [26]**;** the Netherlands | Di Noia et al [25]; United States | Dobbins et al [27]**;** Canada | McVey et al [30]**;** Canada | Sassen et al [28]; the Netherlands | Zhan et al [29]**;** China |
| --- | --- | --- | --- | --- | --- | --- | --- | --- |
| **Brief name** | p. 3  At-Risk in Primary Care | p. 663  Nutrition and Physical Activity Self-Assessment for Child Care (NAP SACC) | p.1  CT e-learning program | p.3  Adolescent substance abuse prevention programs dissemination materials | p. 5  Healthy body weight promotion in children | p. 5  The Student Body: Promoting Health at Any Size | p. 4  Modules on website | p. 2  Online course module |
| **Why** | p. 2  The main objective of this study is to evaluate the effects of  a computer-based role-play simulation on PHPs’ attitudes,  motivation, and behaviours related to mental health screening  and collaborative care of patients who may be experiencing  mental health disorders defined as alcohol and  substance abuse, generalized anxiety disorder, depression,  and posttraumatic stress disorder. | p. 663  The purpose of this study was to determine if web-based training is as effective as in-person training. | p. 1  The aim of the study described here was to assess the effects of  the CT e-learning program on PNs’ smoking cessation guideline  adherence.  Targeting PNs’  behavioural predictors via a Web-based CT support program,  positive behaviour change can be achieved among PNs, meaning  that they improve their smoking cessation guideline adherence. | p. 3  Despite the benefits of using CD-ROM and the Internet for technology transfer, there is a paucity of data on the relative efficacy of these newer technologies over conventional print means of dissemination. The present investigation was undertaken to bridge this gap. | p. 2  To evaluate the effectiveness of a KB in comparison  to other KTE interventions on promoting evidence informed  decision making in public health departments. | p. 5  The overall aim of the present study was to gain an understanding of whether or not the web-based program was a) a tool that teachers and public health professionals felt comfortable or capable using, b) a successful  translation of prevention knowledge over time (e.g., alerting teachers and  public health practitioners to the factors that influence body image), and c)  effective in improving efficacy to fight weight bias. | p. 2  The objective of the Web-based intervention  was to increase health care professionals’ intention and  encouraging behaviour toward patient self-management,  following cardiovascular risk management guidelines. | p. 2  On the basis of the fact that most PHCWs in rural China need more effective training modes to improve their knowledge on BPHS, our study aimed to evaluate the effects of a  blended-learning approach in improving BPHS knowledge  among PHCWs in comparison with a pure e-learning approach. |
| **What** | p. 3  Online professional development  role-play simulation designed to provide PHPs  with simulated conversation experiences where they can  learn to identify and assess patients with mental health  disorders, complete brief behavioural interventions using  motivational interviewing (MI), and refer patients  through collaborative decision making. The simulation  also aims to help PHPs integrate behavioural health into  their treatment and build patients’ intrinsic motivation so  they can better adhere to it. The role-play conversations  were designed to comply with the SBIRT training protocol.  SBIRT itself relies on the use of MI to foster a collaborative  relationship between PHPs and patients to  resolve patients’ ambivalence about changing their  behaviour and bring positive changes for their own health  by highlighting cognitive dissonance between unhealthy  behaviours and healthy goals | p. 663  Each training included the following four modules:  (1) Intervention Overview;  (2) Introduction to Childhood Overweight;  (3) Nutrition and Physical Activity for Children and Adults; and  (4) Providing Consultation to Child Care Centers.  The web training, included interactive features that would mimic components of the in-person training. | p.1  (1) Several  e-learning modules in which PNs had access to individually  tailored advice, a forum, and smoking cessation counselling  materials (both to inform themselves and to provide to smokers)  and  (2) Three general modules with project information,  frequently asked questions about the RCT, and a counselling  checklist to monitor self-reported counselling activities during  the trial. | p. 4  Three youth-oriented substance abuse prevention programs were identified, and illustrative  dissemination materials were developed for each.  To prepare these programs for dissemination, information was synthesized about each, and a common presentation format for delivering this content via pamphlet, CD-ROM, and Internet was developed. Across channels, an overview of the problem of drug abuse was introduced  with a special focus on early adolescents. Discussion was devoted to how drug use is manifested  in their communities, how it affects their lives and futures, and the way in which the nature of  the problem has changed in recent years. Materials described the rationale, strategies, and costs to prevent drug abuse, and the roles of schools, professionals, and community groups, and  relevant private and government bodies in addressing this problem. | p. 5  The least interactive KTE intervention was access to  health-evidence.ca (HE group). Health-evidence.ca is a  repository of all systematic reviews published since 1985 evaluating any public health intervention.  Participants  also had access to the published abstracts, and the full text articles. Finally, a short summary for each of the systematic reviews, written by the research team, identified the key findings and recommendations  for public health policy and practice that were directly applicable to the types of decisions for which the  participants were responsible.  The moderately interactive KTE intervention included tailored,  targeted messages plus access to health-evidence.ca  (TM group). The TM intervention included sending participants a series of emails that included the title of the seven  systematic reviews followed by a link to the full reference, including abstracts, on health-evidence.ca. The online reference offered a link to the short summaries, and finally, the full text of each review.  The most interactive KTE intervention included both the  HE and TM components and a KB who worked one on one with decision makers in the public health departments. | p. 5  The Student Body: Promoting Health at Any Size is an online program  consisting of six learning module topics: Media and Peer Pressure, Healthy Eating, Active Living, Teasing, Adult Role Models, and School Climate. Each  module consists of four steps: (1) A case study intended to  introduce the topic to the facilitator, using a Flash animation cartoon;  (2) Background information designed to provide the facilitator with information  on the topic and its significance to the prevention of disordered eating; (3) Instructions on how to conduct a related classroom activity with students (matched to Ministry of Education learning outcomes); and  (4) Topic-related supplementary resources (e.g., articles, educational videos,  and external sites to explore the topic in more depth). | p. 4 Participants had access to the website, which offered several  modules.  Modules and a forum were  directed at the health care professional to increase professionals’  awareness of their thoughts, and learn skills and strategies to support patients in their own self-management, this to improve their intention and behaviour toward patient-centered health education. The first module enclosed a set of seven screens to  help the professional to improve his or her professional behaviour. The screens contained self-complete forms and were  designed and pre-tested to educate the health care professional,  with a personal feedback system in a “coaching spider chart”.  Next, there was a screen for  planning the encouraging behaviour change, making a plan, and  putting the behaviour change into practice. The third module  consisted of a maximum of seven consultations to encourage  the patient with cardiovascular risk factors, easily adaptable to the needs and individual characteristics of the patients.  The website provided a fourth module with specific information  on physical activity devices, planning physical activity, and  cardiovascular risk factors. The website also included a link to  a forum directed at health care professionals to share experiences  with other professionals in the intervention group. | p. 2  Three course modules were developed based on the BPHS  contents: Course module 1: health management of patients with hypertension; course module 2: health records management  for residents; and course module 3: vaccination. Each course module consisted of 2 parts: theoretical learning and case  studies. |
| **Who Provided** | Not reported | p. 666  Facilitated by the study coordinator | p.2  Web training (no face to face), but facilitated the research team | Not reported | p. 5  The KBs were Master's prepared, had extensive knowledge and  expertise in public health decision making, as well as an understanding of the research process. | Not reported | Not reported | p. 3  Two facilitators were present during the training sessions of both groups for assistance and to answer questions. |
| **How** | p. 3  Online role play simulation | p. 663  In person and web-training | p.1 and 2  E-learning | p. 5  Following receipt of  completed pre-tests, professionals in the respective study arms were sent the pamphlet, CDROM, or logon name, password, and instructions for Internet access. | p. 5  HE group: access to website  TM group: access to website, emails  KB group: access to website, emails and knowledge broker | p. 5 and 6  via an internet browser (online learning modules). | p. 4  Participants had access to the website, which offered several  modules. | p. 3  Control group: via internet modules. presented in the format of Microsoft PowerPoint with 5-6 questions inserted into the slides, and a synchronous audio explanation was  attached in each slide.  Experimental group: Participants in the blended group studied the same  PowerPoint-based theoretical materials available at the same  training platform) during the same period. After that, participants  received the handouts of all case-study materials for  self-studying 4-5 days and attended 1-day (8-h) face-to-face case-study training. |
| **Where** | p. 3  Online in 227 nurses, nurse practitioners,  and doctors from six different states in the US Midwest,  Southwest, and Northeast regions | p. 666  Face to face: Counties across North Carolina for 3 hours  Web-training: provided a URL for access materials | p.2  Across Netherlands (via internet) | p. 3  188 professionals employed in  schools, community agencies, and policy-making bodies in the US., completed online web modules | p. 5  Online for HE and TM group.  KB group: KB who worked one on  one with decision makers in the public health departments. Approximately twenty percent of KB time was spent facilitating  knowledge and skill development either through face-to-face interaction such as workshops or online strategies  such as webinars, interactive web-enabled meetings,  or conferences. Eighty percent of the brokers' time was spent preparing for and directly interacting with participants. | p. 5 and 6  via an internet browser (online learning modules) in two Canadian provinces. | p. 3  Health care professionals in the Netherlands, completed online web modules. | p. 3  The experimental group received  theoretical knowledge on the training platform and the cases  delivered through the face-to-face method. In the control group,  both theoretical knowledge and cases were delivered by the  training platform. |
| **When and how much** | p. 3  The simulation takes between 1 and 1.5 hours to complete  and is self-paced (users can complete the simulation  in multiple sittings), available to each user online 24/7. | p.666  Six in-person trainings were held across the state from December 2005 to March 2006. Each study participant attended one of the six trainings, three hours in length.  Web-training: Participants were asked to complete the web training within three weeks. | p.2  During a 6-month time period  (ie, upon completion of the baseline questionnaire), PNs in the  intervention and control group were free to visit the modules  of the CT e-learning program that were available to them based  on their group allocation as many times as they wanted. PNs could directly print content from the modules and save this  content on their computer. | p. 5  During a 6-month time period. | p. 5 and 6  The three interventions were implemented simultaneously  during 2005.  TM group: Over seven successive weeks, on the same day each week and the same time of day, participants  in the TM group were sent an email indicating that a systematic review related to healthy body weight  promotion in children was available in full text at the link provided.  KB group: The KB initiated  communication with participants occurred at a  minimum of once per month, and more frequently as requested. The KB also offered a site visit to each public health department. | p. 5 and 10  Six modules  Those in the  intervention group had access to the online curriculum for the 60 days  immediately following their completion of the baseline survey. During  the 60-day study period, intervention participants were asked to deliver the online curriculum to students in grades 4, 5, and 6 during regularly  scheduled classroom time. | p. 3  Professionals used the website from January 2011 till June 2012. | p. 2 and 3  The public health services Web-based training  platform based on Moodle was created for the study from August  to October 2013.  All participants were enrolled in the study for an overall period  of 5 weeks (1 week for trainees to familiarize themselves with  training platform; 3 weeks for the theoretical learning; and 1  week for the case study). Before theoretical learning, all trainees could have access to the manual about training platform for 1 week and receive training or guidance for using the platform.  Experimental group: also attended 1 day face to face case study training. |
| **Tailoring** | p. 3  Individualized and immediate  feedback from a virtual coach throughout the simulation. | p.663 The CCHCs training from the NAP SACC intervention was  modified in a number of ways for use in this project.  Nutrition and physical activity recommendations for children were updated, a nutrition and physical activity for  adults section was added, and a number of the small group activities were removed. The overall training was decreased from 5 to 3 h in length.  The in-person and web-based trainings were designed to be similar in both content and structure in order to test  differences in training modality, while holding other factors constant. | p. 2  Individual PNs who were randomly allocated to the intervention  group of the trial had access to all e-learning and general  modules described above and received a tailored feedback letter  based on their answers to the baseline questionnaire; this letter  provided individual PNs with a summary of various pieces of  tailored advice (ie, on different motivational factors and  behavior) and instructions on where to find more elaborate  advice in the e-learning modules. | p. 5  To enhance the relevance and appeal of this content for respondents in each of our target  constituencies, materials were tailored to be responsive to their differing prevention needs. | p. 6  At the start of the intervention, the KB conducted assessments  at the individual, organizational, and environmental  levels, in order to identify strengths, knowledge, and  capacity for evidence-informed decision making. The KB then worked with participants to generate a plan for developing  individual and organizational capacity for evidence-  informed decision making. | Not reported | Not reported. | Not reported |
| **Modifications** | Not reported | Not reported | Not reported | Not reported | Not reported | Not reported | p. 4  The website  underwent no changes during implementation. | Not reported |
| **How well** | p. 7 and 8  When asked for a global rating of the simulation, participants  provided an average rating of 3.12 on a 4-point  scale.  Median  completion time was 155 minutes, while mean completion time was 4,998 minutes | p. 667  Participants in the web training group spent a mean of 124 min (range 53–363 min) on the training modules,  compared to the 180 min spent in training for the in-person group (not including travel time to the training site). All  web trained participants viewed every page of each training module. | Not reported | Not reported | p. 9  It is unknown to what extent the HE group accessed http:/  /www.health-evidence.ca. To our knowledge, all those exposed to the TM intervention received 100% of the intervention. For those exposed to the KB intervention,  approximately 70% received the full intervention (e.g.,  frequency, intensity) with approximately 15%, respectively,  not engaging at all, or to a limited extent. | p. 19-20  Approximately 91% of teachers reported using at least one of the online  modules in the classroom, with nearly half (45.5%) using all six.  Almost all of the participants were satisfied with the selection of module topics (97.3%), as well as the case studies, e.g., Flash animation (90.4%), the  background information for facilitators (98.7%), and the classroom activities for students (89.4%). | p. 8  The module to improve the professionals’ behaviour to optimize processes of shared decision making and  self-management, was used by 45% of the professionals (19/42).  The module with background information on how to coach the  patient with the aim of supporting the health professional in his  or her encouraging behavior toward patients was used by 48% (20/42) of the professionals; The forum directed at improving social support was used by 4 health care professionals. | Not fully reported.  p. 4  In total, 105 participants  were lost to follow-up in course module 1; 95 in course module 2; 124 in course module 3 in the experimental group; and 87,  84, and 78 participants were lost to follow-up in course module  1, module 2, and module 3, respectively, in the control group. |

Page numbers have been provided for the original primary journal articles, where available
